# Spectral Relative Attenuation of Solar Radiation through a Skylight Focused on Preventive Conservation: Museo De L’almoina in Valencia (Spain) Case Study

**DOI:** 10.3390/s21144651

**Published:** 2021-07-07

**Authors:** María-Antonia Serrano, José-Luis Baró Zarzo, Juan-Carlos Moreno Esteve, Fernando-Juan García-Diego

**Affiliations:** 1 Centre for Biomaterials and Tissue Engineering, Universitat Politècnica de València, Camino de Vera, s/n, 46022 Valencia, Spain; mserranj@fis.upv.es; 2 Department of Architectural Composition, Universitat Politècnica de València, Camino de Vera, s/n, 46022 Valencia, Spain; jobazar@cpa.upv.es; 3 Department Applied Physics, Universitat Politècnica de València, Camino de Vera, s/n, 46022 Valencia, Spain; jcmestev@fis.upv.es

**Keywords:** preventive conservation, archaeological heritage, solar irradiance, transparency, glazing, UVB band, UVA band, VIS band, NIR band, spectrometer

## Abstract

The aim of the present study was to evaluate the relative attenuation of VIS, UV and NIR solar radiation through a large pond skylight into the interior of the l’Almoina Archaeological Museum (Valencia, Spain), and to determine how relative attenuation varied throughout the year and time of day. Measurements were taken at 9:00 a.m., 12:00 p.m. and 3:00 p.m. during July 2019 and January 2020. Relative attenuation values were obtained from the measurement of spectral irradiance in the exterior and at different points in the interior by means of two Ocean Optics spectrometers: HR4000CG-UV-NIR for VIS (400–700 nm) and NIR (700–1000 nm) bands, and FLAME-S-UV-VIS for UV-A (280–315 nm) and UV-A (315–400 nm) bands. The central points of the skylight had relative attenuation at 520 nm, reaching a value of 50% in summer at noon and 38% in the afternoon. At noon in winter, there were two relative attenuation peaks above 33% at 520 nm and at 900 nm. For mean relative attenuation, in the UVB range, the highest relative attenuation (20%) was inside the ruins in the morning in both summer and winter, and the UVA band relative attenuation was quite constant throughout the museum, but lower than that of the UVB band, in the range 0–3%.

## 1. Introduction

The preventive conservation and maintenance of archaeological sites is one of the requirements of the main international charters on the protection of archaeological heritage [1,2,3,4]. Protection against excessive light radiation and ultraviolet rays (UV), in particular, is one of the factors in the proper conservation of cultural assets [5] (pp. 158–164).

Indeed, prolonged exposure to visible light (VIS) is known to cause pigment discoloration, and UV causes yellowing and disintegration of certain materials, while the infrared band (IR) causes warming of the surface of objects that can eventually lead to thermal deterioration [6].

The case study described here is within an archaeological site in Valencia (Spain) containing the remains of different historical epochs, the oldest of which dates back to the foundation of the city in the Roman Republic period [7,8]. The site is next to two of the most representative of the city’s religious buildings: Cathedral and the Basilica of Our Lady of the Forsaken. A museum was built in 2006 to preserve the site, on which a public square was erected, including a large skylight of about 300 square meters (approximately 17 × 17 m) above the old thermal baths.

This decision made it possible to capture intense solar lighting throughout the day and, at the same time, an effect of transparency that attracts the curiosity of the passers-by and suggests, from the inside, the presence of an open space. The skylight consists of a three-leaf laminar glass plate covered by a thin sheet of water, producing a quivering effect of the projected light [9]. The interior lighting of the museum and its general atmosphere depend, to a large extent, on the skylight, even though it only covers 12% of the museum’s roof.

Although the museum and the public space generated have received a good reception from the public, the truth is that the presence of the skylight is causing significant practical problems. In addition to the extensive maintenance required by the need for cleaning and the appearance of leaks, other problems are no less serious, such as the thermal oscillation, the generation of condensation or the drying effect that favors the formation of efflorescence [10]. On the other hand, the environment generated is conducive to biological growth of microorganisms, vegetation and insects, and in summer, there is low energy efficiency due to the greenhouse effect [11], while there is a strong contrast in light intensity between the areas under the skylight and under the opaque roof.

These setbacks caused a reaction in public decision makers, who proposed replacing the pond with a pyramidal skylight in the style of the Louvre in Paris [12], or alternatively the removal of the water sheet [13], although, thus far, neither of these proposals has been carried out [14]. In our opinion, the removal of the water sheet would make temperature control more difficult, in agreement with Fernández-Navajas et al. [15] and Merello et al. [16].

The scientific literature provides several analyses on the conservation of archaeological sites exposed to solar radiation. Funda Yaka [17] considered the undoubted advantages of transparent protection but warned of the disadvantages and the need to carefully select the material according to its thermo-physical properties. In fact, the consequences of an excess of radiation were the trigger for the removal of the transparent protective structures of the Roman Villa at Piazza Armerina in Sicily (Italy) and the Fishbourne Roman Palace at West Sussex (England). Michalsky [6,18,19] recommended adjusting illuminance conditions by balancing the compatibility between the visibility necessary for minimal artistic fruition and the low vulnerability necessary for conservation.

Al-Obaidi et al. [20] warned of the need to control UV with laminated glass, the most commonly used glass type in museum skylights, especially in the case of larger opening areas, where a lower visible transmittance and solar heat gain coefficient are required. For his part, Horie [21] dealt with the use of solar control films to reduce light levels in buildings with daylight without altering either the external or internal appearance of the whole. According to Camuffo [5], the simple physical effect of light absorbed in the form of heat will affect the surface temperature and RH and may induce internal stress. The humidity and temperature conditions inside the l’Almoina Museum have, in fact, been treated in recent specific studies [15,16].

The type and thickness of the glass used in the skylight have a significant influence on the UV spectrum. Several authors [22,23,24,25] found that glass filters out the UVB band of solar radiation but transmits a large part of the UVA band.

Tuchinda et al. [25] analyzed factors that could have affected glass’s UV-protective properties, including glass type, color, interleaves and coating, among others. They indicated that clear glass allowed up to 72% of UV light and up to 90% of VIS to pass through, depending on the thickness of the glass.

Li et al. [26] studied the optical performance of glazing units in the UVA, PAR and NIR bands, and they obtained a small transmittance difference spectrum between different thicknesses of single glazing units in the VIS range (380–760 nm), but a large difference in the UV and NIR bands, reducing transmittance as the thickness increased. They also found that transmittance in the VIS region of a quartz glass slab was higher than 78%, due to the absorption band, and between 40% and 80% in the UVA region.

Serrano and Moreno [27] studied the spectral transmission of solar radiation by materials and, for smoked glass, found transmittance values ranging between 56% and 68% in the UVB band and 70% in the UVA range, with higher values in the VIS (85%) and NIR (80%) ranges; for smoked glass, they obtained lower transmittances at higher temperatures, which could be due to the reduced thermal conductivity when the temperature rises [28].

In the previous works cited [25,27] and in others [29,30], laboratory experiments were performed to measure the transmittance as defined in [31]. In the present work, this same procedure was used to measure the maximum light intensity that affects a point, fundamental data for the preventive conservation of heritage as proposed by the standard [32]. The relationship between the outside incident lightning at a window and the maximum incident lightning at an inside point is not defined in the bibliography [31], and therefore it is called “relative attenuation” in this work.

The present study aimed to evaluate the relative attenuation of solar radiation in the interior of the l’Almoina through the water-covered skylight, for VIS, UV and NIR, and to determine how relative attenuation varies over time, throughout the day and the year; how it is distributed in space; and to what extent it is affected by the presence of the sheet of water over the glass, all this taking into account that the l’Almoina is a museum and the skylight was designed mainly for lighting and visual relationships, but not for the ideal environmental conditions for the conservation of the archaeological remains. In this regard, the study joins other studies already published that analyzed temperature and humidity variables such as those mentioned above.

## 2. Materials and Methods

### 2.1. Tested Materials

**Figure 1:**
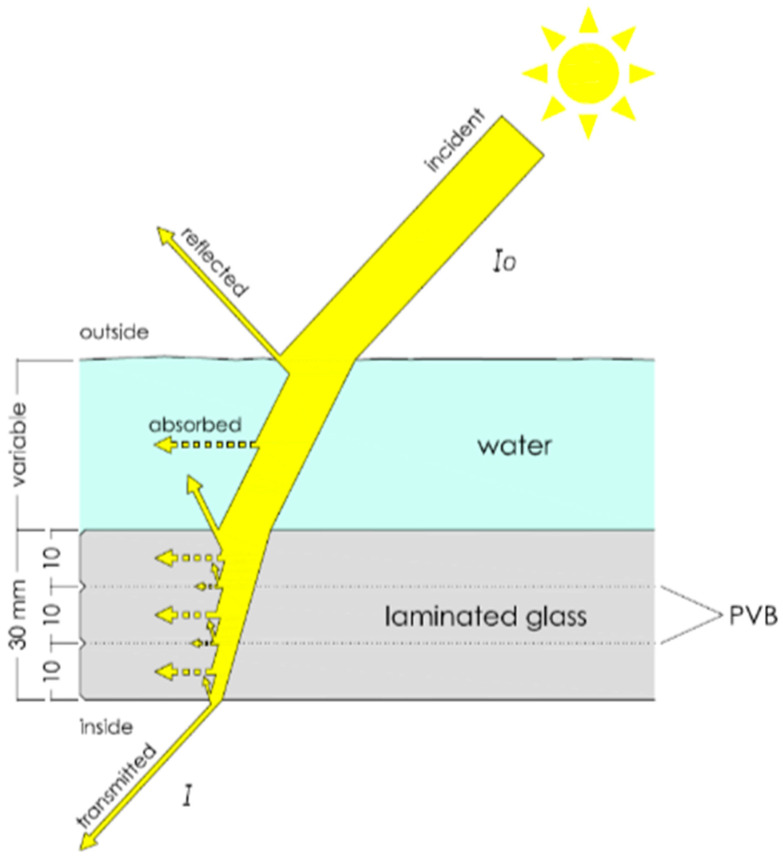
Diagram of optical relative attenuation through skylight and water layer. The initial intensity (*I*_0_) is reduced by the partial absorption and reflection experienced when passing through the different layers, meaning that only a fraction is transmitted to the interior (*I*).

This study took place in the Museo de l’Almoina, in the historic center of Valencia (39–28′ N, 0–22′ W, at sea level), on the Spanish east coast. The element on which the research was based is a large skylight composed of a laminated glass consisting of 3 sheets of 10 mm-thick glass joined together by sheets of polyvinyl butyral (PVB) (Figure 1). This material was used to increase the flexural strength of the glass based on the adhesion capacity of the sheets and also for safety factors in case of breakage, since it prevents shattering. In addition, it contributes to reducing the relative attenuation of UV radiation, as we found in this study.

### 2.2. Spectral Measuring Devices

Relative attenuation values were obtained from the measurement of spectral irradiance in the exterior and at different points in the interior by means of two Ocean Optics spectrometers [33]: HR4000CG-UV-NIR for VIS (400–700 nm) and NIR (700–1000 nm) bands, and FLAME-S-UV-VIS for UV-A (280–315 nm) and UV-A (315–400 nm) bands. The reason for the duplication was due to the different working ranges and calibration to which they were submitted at the time, as we shall see later.

**Figure 2:**
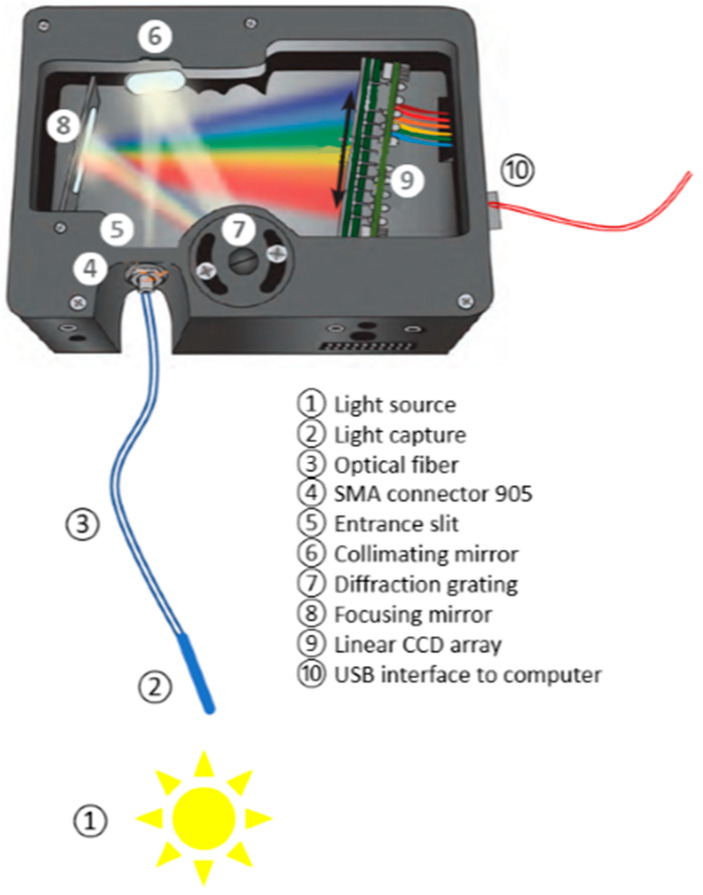
Operating diagram of an optical spectrometer such as the one used in this study [34].

The operation of both devices is similar (Figure 2), although with different technical specifications (Table 1). Light is captured and passes through a fiber-optic cable to the spectrometer using an SMA connector. The entrance slit regulates the amount of light entering the optical bench, and the filter regulates the predetermined wavelength region. Light is reflected in the collimating mirror towards the grating, where it diffracts. Then, it is directed to the focusing mirror from which it is projected to the CCD (charge-coupled device) to convert optical information into a digital signal. The spectrometer transmits the digital signal from the USB port to the computer, where it is managed by the software.

Both spectrometers were calibrated in July 2017 from 250 to 1000 nm and from 250 to 400 nm for HR4000CG-UV-NIR and FLAME-S-UV-VIS, respectively, by Ocean Optics, with a measurement uncertainty of approximately 10% across the entire measurement spectrum.

### 2.3. Data Collection

The capture and reception management of the information provided by the two spectrometers was performed on a conventional PC using the applications OOIBase32 (for HR4000CG-UV-NIR) and SpectraSuite (for FLAME-S-UV-VIS), both by Ocean Optics.

**Figure 3:**
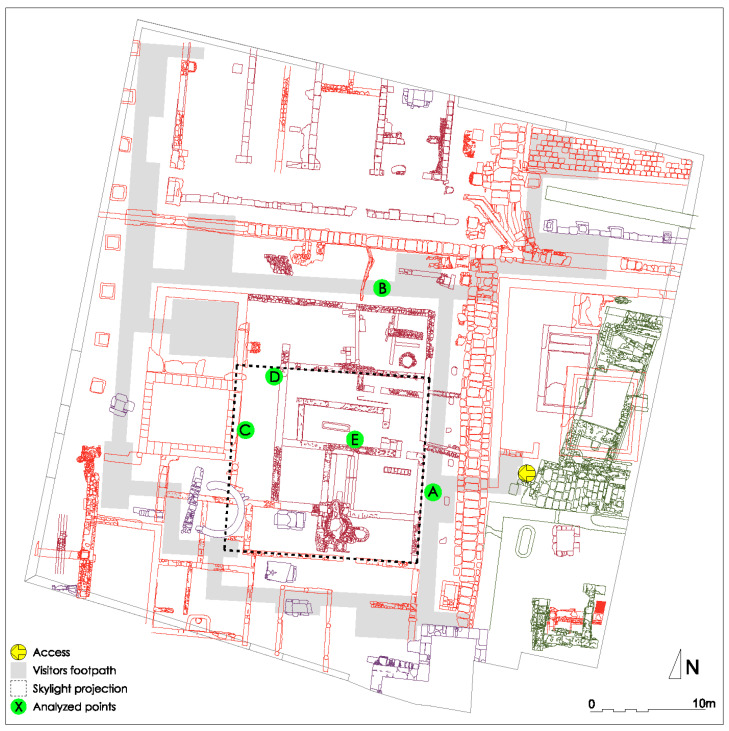
Spatial distribution of recording points [35].

The sensor was first directed towards the maximum light intensity point that coincides with the solar position to record normal solar irradiance, and immediately afterwards, the measurement was taken in the same way at a total of 17 points just below or around the skylight projection (Figure 3). Two successive records were taken from each point to rule out possible errors. For further operational calculations, five of these points were subsequently selected: point A, next to the landing of the entry staircase; point B, on the right-hand corridor; point C, on the ground; and points D and E under the skylight.

This procedure was repeated on different days of the year: two in summer (25 and 30 July 2019) and one in winter (14 January 2020). In the case of 25 July, the skylight did not have the water sheet on its upper surface, while on 30 July, it did, which is the usual situation except during maintenance and cleaning operations. On 14 January, the skylight was filled with water. The procedure was also repeated at different times of the day: 9.00 a.m., 12.00 p.m. and 15.00 p.m. (solar time). The three days when the spectral values were taken were clear. The solar zenith angle on the data collection days is shown in Table 2.

### 2.4. Study Range

**Figure 4:**
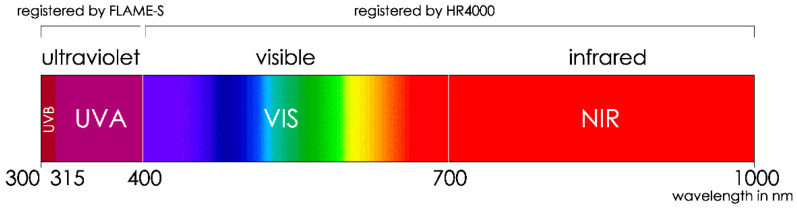
Spectral bands and corresponding wavelengths.

As the UVB radiation values below 300 nm with FLAME-S had a lot of background noise, they were discarded from the calculations. Wavelengths above 1000 nm were also disregarded for the same reason, meaning that the total range covered was from 300 to 1000 nm. This range was organized into UVB, UVA, VIS and NIR bands, according to their physical properties and their environmental effects (Figure 4).

### 2.5. Calculation of Relative Attenuation

When light reaches a semi-transparent surface, part of it is reflected, part of it is absorbed and part of it is transmitted through the object (Figure 1). This phenomenon can be expressed by the following irradiance balance that can be particularized for each wavelength:

*Irrad_incident,λ_* = *Irrad_reflected,λ_* + *Irrad_absorbed,λ_* + *Irrad_transmitted,λ_* (1)

Optical relative attenuation, as the main magnitude on which this entire study pivots, defines a relative—dimensionless—quantity that measures the fraction of incident light passing through a sample (2), in this case, the glass of the skylight. It is expressed by the result of dividing the transmitted light maximum intensity (*Imax*) by the incident maximum ray intensity (*I*_0*,max*_).

*Relative attenuation* = *Imax / I*_0,*max*_ (2)

The calculations for obtaining the relative attenuation values were based on the data provided by the spectrometers (*Imax*, *I*_0*,max*_).

In order to evaluate the comparative incidence of the different spectral bands for a given point, time and day, we needed to calculate an average value for each wavelength range studied (UVB: 300–315; UVA: 315–400; VIS: 400–700; NIR: 700–1000) according to the following expression:

(3) $$\frac{\int^{\;}I_{\lambda }dy}{\int^{\;}I_{o\lambda }dx}$$

where *I_𝜆,max_* is the irradiance transmitted through the skylight, and *I*_0_*_𝜆,max_* is the normal solar irradiance for the same wavelength range. In our case, the step provided by the spectrometer, which is approximately 0.10 nanometers, was taken as the wavelength increment for the purpose of the calculations.

### 2.6. Comparative Relative Attenuations

According to the objectives set when planning the study, data management was specific in each case:

- To evaluate the influence of irradiance due to the different sun inclinations throughout the year, the relative attenuation data of a summer and a winter day were compared for the same hours (9, 12 and 15 solar hours) and the same points;
- To evaluate the influence of irradiance due to the different orientations and inclinations of the sun throughout the day, the relative attenuation calculations were compared at different times (9, 12 and 15 solar hours) for the same season and same points;
- To compare the relative attenuation of the skylight with and without water in the different frequency bands, we worked with the same sampling period—summer—with data from two days close together when the skylight was dry for maintenance (25 July 2019) or with the water sheet (30 July 2019);
- To take into account the spatial variations in relative attenuation inside the museum, the spectral values of all the points recorded by the UV and VIR-NIR bands were considered for the same season, the same time and the same skylight situation (clear days without clouds).

The selected data were arranged in tables and presented in graphs using Microsoft Office Excel for a clear visualization of the results.

## 3. Results and Discussion

Regarding the precision of the measurements, the calibration uncertainty of 10% is the same as that with which other authors have worked [25,27,29,30]. We carried out the same measures under real conditions with a similar instrumentation to verify their effectiveness and usefulness in preventive conservation of cultural heritage [32]. This could be conducted due to the great repeatability of the instruments used [33].

### 3.1. Measured Relative Attenuation Discussion

In this work, we measured the relative attenuation that does not correspond to the accepted definition of transmittance [31]. Transmittance was measured experimentally under laboratory conditions [25,27,29,30] by measuring the intensity of the incident and transmitted rays with directional devices.

The measurement performed in this work is the minimum attenuation at a point, since it is the relationship between the maximum incident intensity of the upper part and the maximum at a lower point.

This coincides with the norm [32] for the conservation of cultural heritage in terms of lighting. This standard [32] proposes measuring the maximum illumination to which a work is subjected and, if it is composed of several materials, the material most sensitive to light.

The methodology proposed in this work is useful in museums with natural lighting since, as in this case overhead, the maximum intensity at a point depends on many architectural factors, and the only way to know it is to precisely measure it.

According to the standard [32] for a stone museum, lighting is not a factor that affects the preventive conservation of the museum. Nevertheless, it must be taken into account that in this museum, there are display cabinets with various valuable objects, and that they can change if considered by the museum directors. It is also a space capable of hosting temporary exhibitions. Therefore, the quality and quantity of lighting really matters for cultural heritage preventive conservation [32].

### 3.2. Spatial Study of the Spectral Relative Attenuation of the UV, VIS and NIR Bands

#### 3.2.1. UV Band

**Figure 5:**
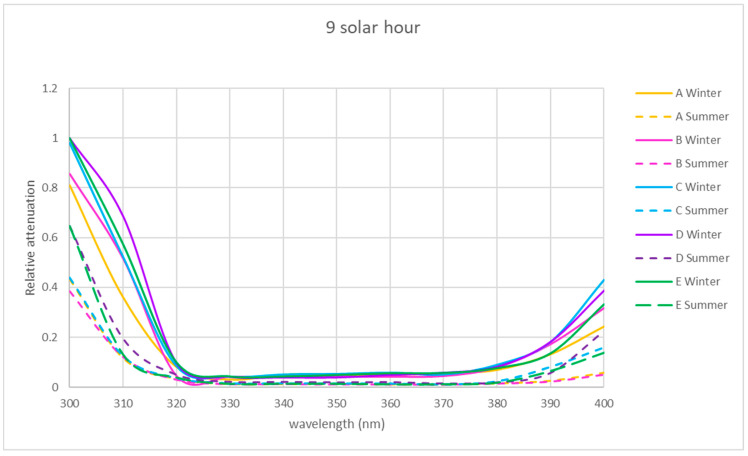
Representation of the spectral relative attenuation for the UV band for the 5 locations at 9 a.m. solar time.

**Figure 6:**
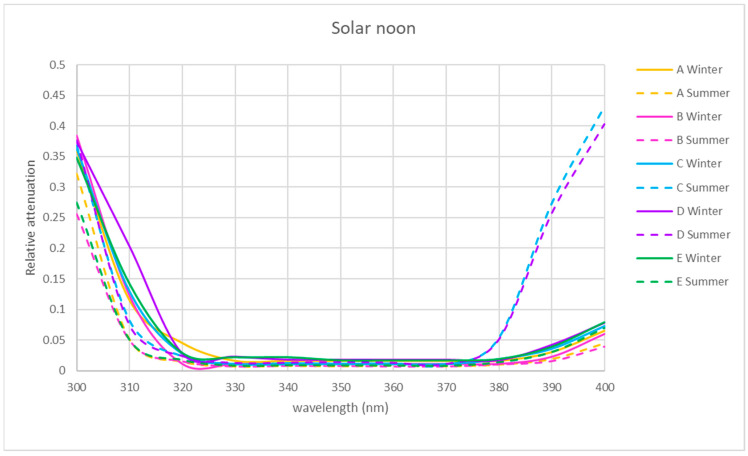
Representation of the spectral relative attenuation for the UV band for the 5 locations in Scheme 7. Representation of the spectral relative attenuation for the UV band for the 5 locations studied in the two seasons of the year at 15 h solar time.

**Figure 7:**
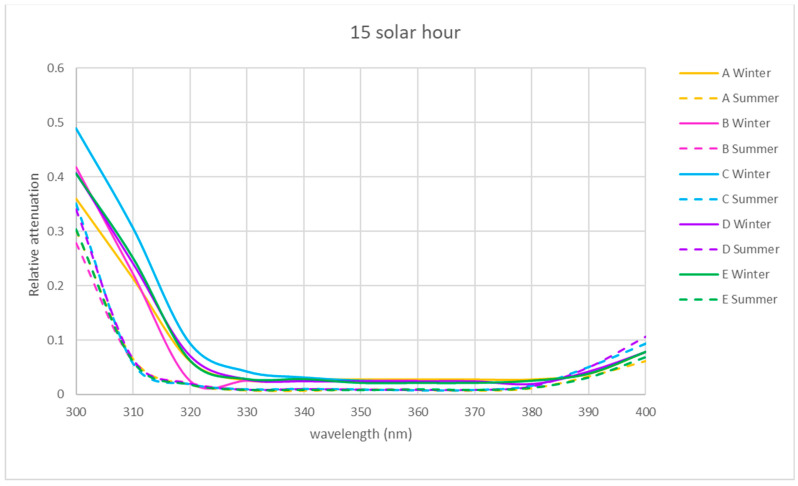
Representation of the spectral relative attenuation for the UV band for the 5 locations studied in the two seasons of the year at 15 h solar time.

The spectral relative attenuation of the UV band was calculated according to Equation (2) for the chosen sites and two seasons of the year and is represented graphically in Figure 5, Figure 6 and Figure 7 for the three times of the day studied: 9 a.m. (morning), 12 p.m. solar (noon) and 15 p.m. (afternoon).

Figure 5, Figure 6 and Figure 7 show that for the physical locations studied, the general trend is similar. The relative attenuation is at its maximum at 300 nm (80% on the walkway (A)) and almost 100% for locations within the ruins (C and D), and it decreases to 320 nm, where it is at its minimum, from 370 nm. The relative attenuation values then rise slightly again, reaching 40% for locations C and D and 25% for location A.

These higher relative attenuations at 300 nm reach their highest values in winter in all the locations. In this season, at 300 nm, the relative attenuation value observed at all locations was over 80% (Figure 5) in the morning, ranging in the afternoon between 35 (location A) and 50% (location C) (Figure 7), and at noon, the relative attenuation was at its minimum (about 35%) for all locations (Figure 6).

In summer, the general trend was also similar in all locations. At 300 nm, the relative attenuation was also at its maximum in the morning, around 60% in location D and about 40–45% at the rest of the locations (Figure 5), ranging in the afternoon between 35 and 40% (locations C and D) and 25 and 30% (rest of locations) (Figure 6 and Figure 7), and it was at its minimum at noon (25 to 35%) for all locations.

It is observed for the UV band that the relative attenuations are lower at noon (Figure 6) than at 9 h or 15 h (Figure 5 and Figure 7). This could be attributed to the fact that the temperature increases towards noon, which could produce an electronic delocalization of the material composing the skylight, which can lead to an increase in harmonic resonances, and therefore a decrease in the relative attenuation [36].

It is also observed in each of Figure 5, Figure 6 and Figure 7 that for each hour of the study, the relative attenuation is slightly higher in winter than in summer, also attributable to the lower temperature in winter.

#### 3.2.2. VIS and NIR Bands

**Figure 8:**
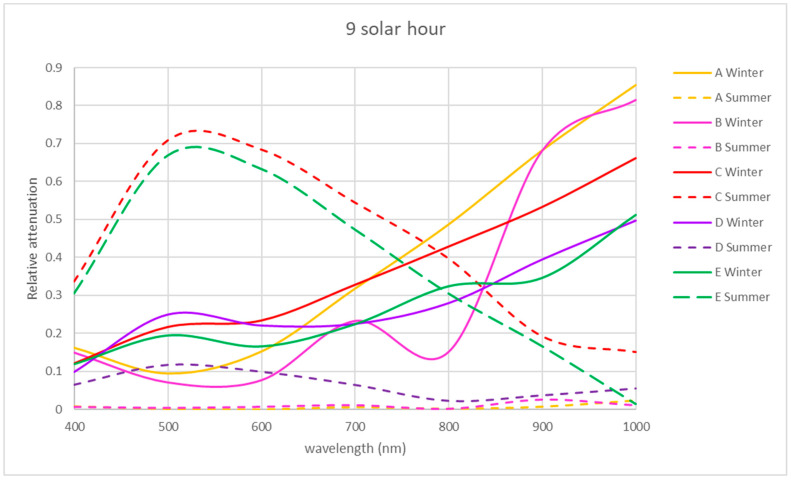
Representation of the spectral relative attenuation for the VIS and NIR bands for the 5 locations studied in two seasons of the year at 9:00 a.m. solar time.

**Figure 9:**
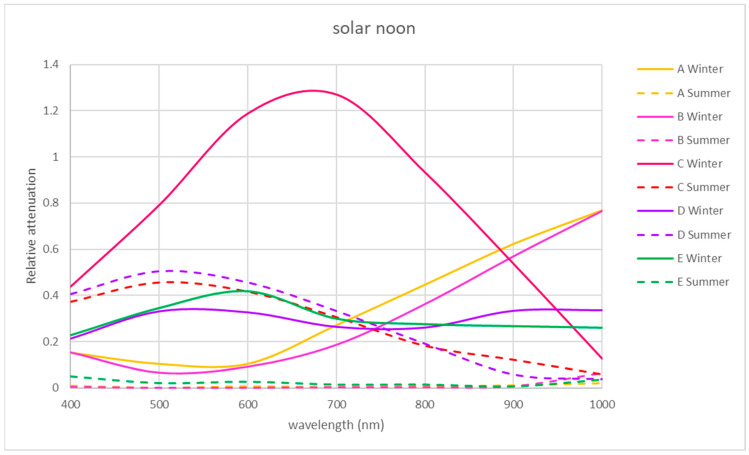
Representation of the spectral relative attenuation for the VIS and NIR bands for the 5 locations studied in two seasons of the year at solar noon.

**Figure 10:**
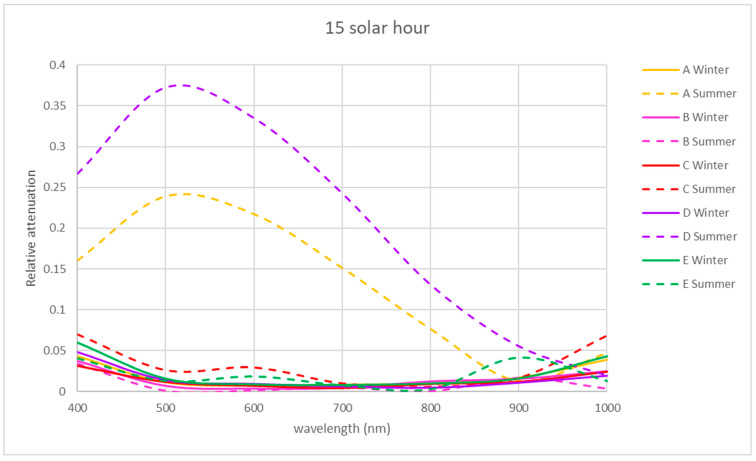
Representation of the spectral relative attenuation for the VIS and NIR bands for the 5 locations studied in two seasons of the year at 15 solar hours.

For the VIS and NIR bands, the same procedure was followed as for the UV band using Equation (2) (see Figure 8, Figure 9 and Figure 10).

The winter morning and noon relative attenuations reveal wide variability, as shown in Figure 8 and Figure 9. In winter, in the morning, the relative attenuation shows an upward trend from 400 to 1000 nm for all the locations studied, as shown in Figure 8, reaching 60% at 1000 nm at location C and 50% at locations D and E.

NIR relative attenuation at locations A and B is significant in winter in the morning and at noon (Figure 8 and Figure 9), with a value of 30% at 400 nm, reaching 80% at 1000 nm. Location C showed a similar tendency in the winter morning but reached a relative attenuation maximum value of 70%.

In the NIR band, locations A and B presented a similar tendency to the rest of the studied periods, with a relative attenuation below 15%.

In the VIS band, locations A and B had a similar tendency during all the studied periods, with a relative attenuation value below 5%, except for location A in summer afternoons (25%).

Exceptionally, at location C, there was a greater transmission in winter at midday, exceeding 100% at 670 nm. In this location at the VIS band in summer, the relative attenuation values reached 70% and 50% in the morning and noon, respectively (Figure 8 and Figure 9). In the VIS and NIR bands in the afternoon in location C, the relative attenuations are negligible, as in locations A and B. In the area of location D, there is greater relative attenuation at 520 nm, reaching a value of 50% in summer at noon (Figure 9) and 38% in the afternoon (Figure 10). In winter at noon, as shown in Figure 9, there are two relative attenuation peaks above 33%, at 520 nm and at 900 nm.

As seen in Figure 8, the highest relative attenuations in location E occurred in the morning, reaching 70% at 520 nm in summer and 50% at 1000 nm in winter. At midday in winter, attenuation reached 40% at 600 nm (Figure 9).

### 3.3. Hourly Study of the Spectral Relative Attenuation of the UV, VIS and NIR Bands

#### 3.3.1. UV Band

In the two seasons of the year studied, the shape of the relative attenuation curve is similar for all the locations studied, as it can be seen in Figure 5, Figure 6 and Figure 7. The curves present a maximum at 300 nm, decrease to 320 nm, remain negligible up to 370 nm and slightly increase in the morning and afternoon, with a greater increase at midday in summer for interior locations C and D, reaching a relative attenuation of 45%.

In the morning and at solar noon, the highest relative attenuation (almost 100% in the morning and 40% at solar noon) occurred at 300 nm at locations C, D and E in winter, as shown in Figure 5 and Figure 6.

At solar noon, as shown in Figure 6, at 400 nm, the highest relative attenuation occurred in summer at locations C and D, reaching 45%.

In the winter in the afternoon, as it can be seen in Figure 7, location C presents higher relative attenuation values (50%) at 300 nm, and locations B, D and E present similar values (40%).

#### 3.3.2. VIS and NIR Bands

The trend found in winter in the morning regarding the increasing relative attenuation from 400 to 1000 nm at all the locations studied can be seen in Figure 8.

At 9 solar hours, in the NIR band in summer, the relative attenuation dropped from 50 to 5–10%, as shown in Figure 8, unlike in winter, when it increased from 20–30% until reaching 50–80%. This happened at all locations. In the VIS band, the maximum relative attenuation (70%) was reached at 525 nm in summer at locations C and E.

At solar noon (see Figure 9), there were non-uniform trends in the relative attenuation variation in winter: in some locations (A and B), it rose, while in others (D and E), it varied little, and in location C, the relative attenuation was greater than 100%. In summer, the relative attenuation was very small at locations A, C and E.

At 15 solar hours, the relative attenuation was quite small in the two seasons studied at all locations, as it can be seen in Figure 10, except for locations A and D in summer, where maximum relative attenuations of 25 and 37% were reached, respectively, at about 510 nm.

### 3.4. Mean Relative Attenuation of UVB and UVA Bands

**Figure 11:**
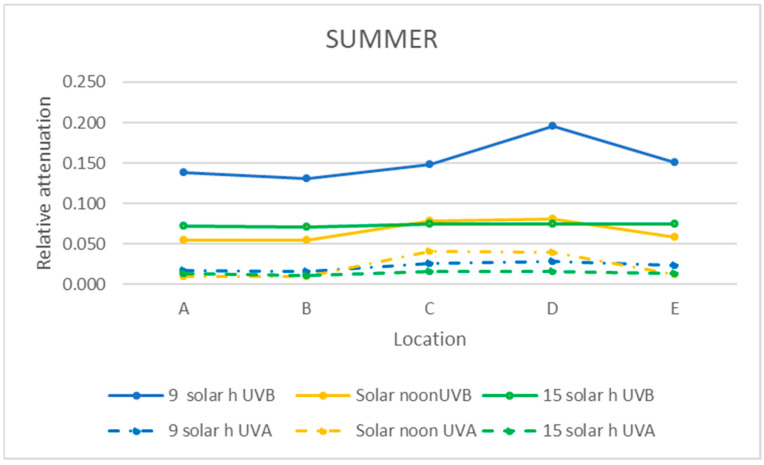
Representation of the mean relative attenuation for the UVB and UVA ranges for the 5 locations studied in summer at three times of day.

**Figure 12:**
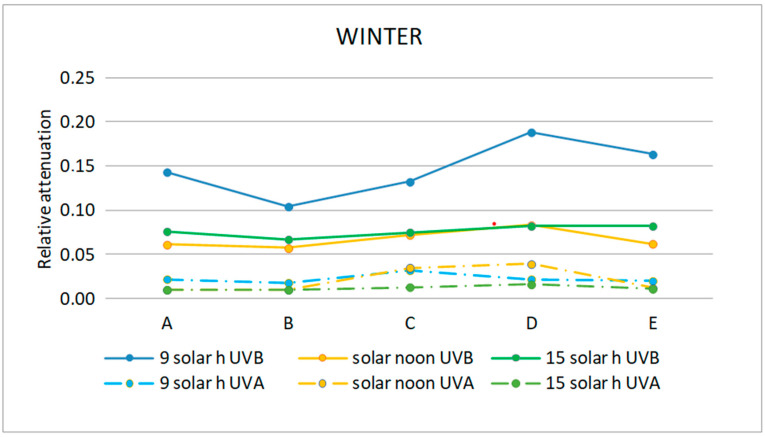
Representation of the mean relative attenuation for the UVB and UVA ranges for the 5 locations studied in winter and at three times of day.

The relative attenuation of the UVB and UVA bands was calculated using Equation (3) and is shown in Table 3. The graphic representation of the two seasons of the year studied is shown in Figure 11 and Figure 12.

In the UVB range, the highest relative attenuation (20%) is inside the ruins (locations D and E) in the morning in both seasons, as shown in Table 3 and Table 4. In this range, at noon and in the afternoon, the relative attenuation remained fairly constant throughout the museum, between 5 and 10%.

As it can be seen in Figure 11 and Figure 12, the relative attenuation of the UVA band is also quite constant (<−3%) throughout the museum in both seasons, but lower than that of the UVB band.

### 3.5. Mean Relative Attenuation of VIS and NIR Bands

**Figure 13:**
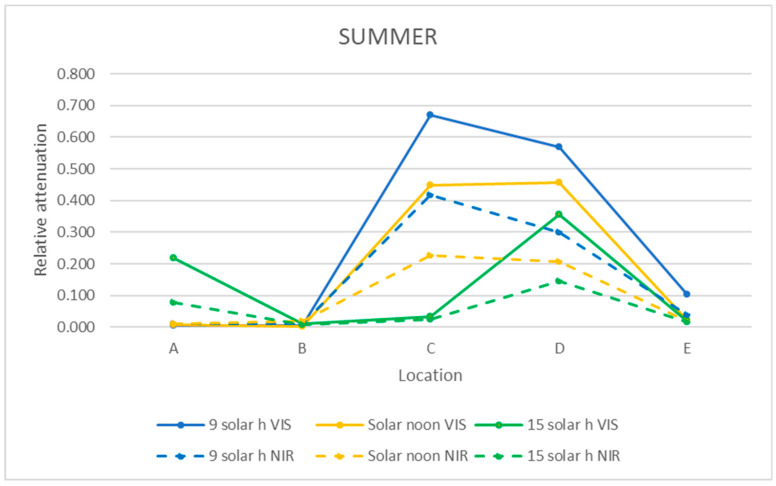
Representation of the mean relative attenuation for the VIS and NIR ranges for the 5 locations studied in summer at 3 times of day.

**Figure 14:**
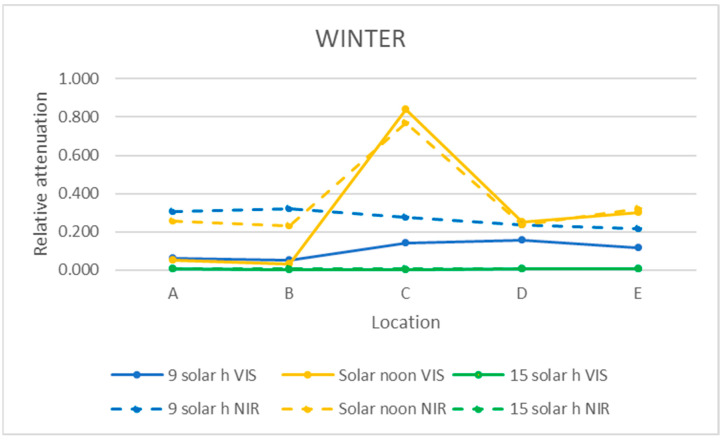
Representation of the mean relative attenuation for the VIS and NIR ranges for the 5 locations studied in winter at 3 times of day.

The relative attenuations obtained following the same procedure as for the UV band (Equation (3)) are shown in Table 5 and Table 6 and graphically represented in Figure 13 and Figure 14.

In summer in the VIS and NIR bands, the relative attenuation was very small at all times on the walkway (locations A and B), except in the afternoon in the VIS range, which reached 22% in location A, as it can be seen in Table 5. The highest relative attenuation occurred within the ruins (locations C and D), as shown in Figure 13, and decreased from morning to afternoon, but with lower relative attenuation values in the NIR range (morning 35%, afternoon 15%) than in the VIS range (morning 50–65%, afternoon 30%).

In winter, on the walkway (A and B), the relative attenuation is very small at all times in the VIS range and in the afternoon in the NIR range. In both ranges, the highest relative attenuation reached the maximum value (85%) at noon within the ruins (location C).

In the morning, the relative attenuation was higher in the NIR range than the VIS range (30% vs. 10%), presenting little variation throughout the entire museum.

In the afternoon, in both ranges, the relative attenuation was less than 1% at all of the studied locations.

Analyzing Figure 8, Figure 9 and Figure 10 of the VIS and NIR bands, a great variability is observed, although analyzing Figure 13, where the mean attenuations for both bands are represented, a higher relative attenuation is also observed in the early morning. On the other hand, in winter (Figure 14), the same behavior does not occur.

This fact can also be verified in Figure 11 and Figure 12 for the UVB band, while in the UVA band, the relative attenuations are low (less than 5%), and there is no appreciable difference between moments of the day studied.

### 3.6. Comparison of UV Spectral Relative Attenuation for Skylight States with and without Water Sheet

**Figure 15:**
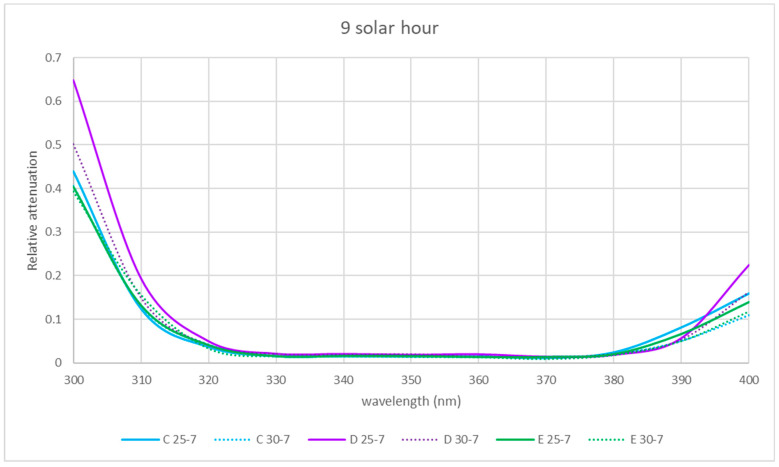
Comparison of spectral relative attenuation for the UV band for the three interior locations for the skylight without (25-7) and with water (30-7) at 9 a.m. solar time.

**Figure 16:**
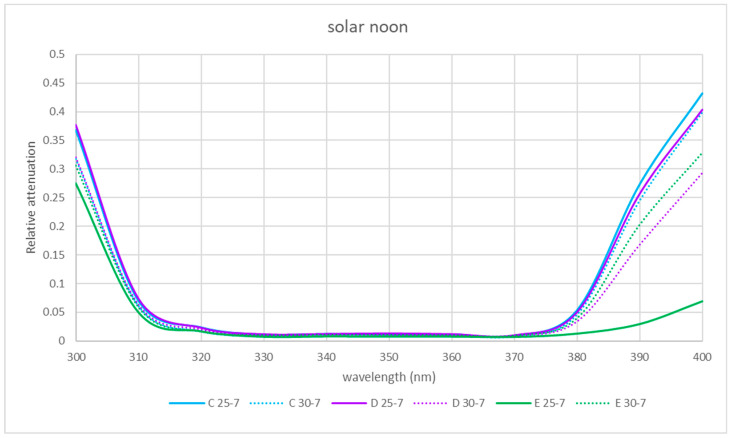
Comparison of spectral relative attenuation for the UV band for the three interior locations for the two skylight states (25-7 without water and 30-7 with water) at solar noon.

**Figure 17:**
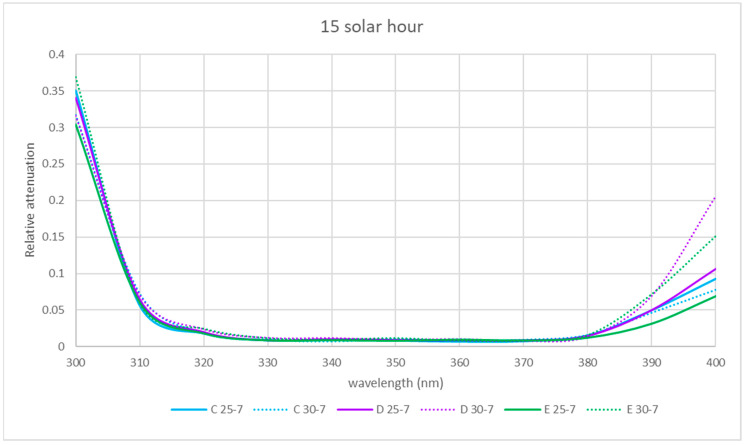
Comparison of spectral relative attenuation for the UV band for the three interior locations for the two skylight states (25-7 without water and 30-7 with water) at 15 solar hours.

The spectral relative attenuation was calculated according to Equation (2) for the locations studied and the UV band (see Table 7, Table 8 and Table 9). The interior locations (C to E) are represented as the most significant and are shown in Figure 15, Figure 16 and Figure 17.

At 9 solar hours and solar noon, the relative attenuation was generally higher when the skylight was without water (day 25-7), except for location E at noon. At locations C, D and E, in the afternoon, relative attenuation was higher from 380 nm when the skylight had no water.

## 4. Conclusions

This study of the relative attenuation of the UVB band from 300 nm and the UVA, VIS and NIR bands through the skylight indicates that the highest relative attenuation occurs in the VIS band (range 70–80%) in both seasons and in the NIR band (80%) in winter. Our results are in agreement with those of Tuchinda et al. [25], who found that clear glass allows up to 90% of VIS light to pass through, depending on its thickness, and with those of Li et al. [26], who found relative attenuations higher than 78% in the VIS region of a quartz glass slab. Additionally, our results also agree with Serrano and Moreno [27], who found relative attenuation values of 85% in the VIS band and 80% in the NIR range for smoked glass.

In the UV range, the skylight relative attenuation is higher in the UVB band than in the UVA band (20% vs. 5%). These results do not agree with the conclusions reached in other studies, such as Tuchinda et al. [25], Li et al. [26] and Serrano and Moreno [27], who obtained UVA relative attenuations in glass ranging between 40 and 80%. These different results could be due to the fact that the skylight had several layers of glass with PVB and a sheet of water on the surface.

The observed results of a lower relative attenuation at higher temperatures, as happens at noon, could also be due to the fact that when the temperature increases, the thermal conductivity decreases, which leads to a greater thermal inertia. In conclusion, this would lead to a lower relative attenuation.

Summarizing, we found that summer relative attenuations were higher at 9 h solar than at solar noon and were similar to those in winter. This could be due to the higher summer thermal level at solar noon that could cause the network of elastomers to rotate and vibrate, intercepting most of the solar radiation incident photons. Additionally, the mechanical properties of the materials could produce this, considering that thermal diffusivity is higher at lower temperatures and thus relative attenuation is increased. When the temperature is higher, diffusivity is reduced [36], and therefore relative attenuation is decreased.

The sequence of this study was as follows: measurements were made at specific points of the museum of UV, VIS and NIR bands at certain times, and a maximum at 300 nm and a minimum at 370 nm were detected as a common result.

The VIS and NIR bands around location D (one of the central points) showed greater relative attenuation at 520 nm, reaching a value of 50% in summer at noon (Figure 9) and 38% in the afternoon (Figure 10). As it can be seen in Figure 9, in winter, at noon, there were two relative attenuation peaks above 33%, at 520 nm and at 900 nm.

At location E (central point), the highest relative attenuations occurred in the morning, reaching 70% at 520 nm in summer and 50% at 1000 nm in winter. At midday, in winter, they reached 40% at 600 nm (Figure 9).

For the VIS band, the maximum relative attenuation (70%) was reached at 525 nm in summer at locations C (south point) and E (central point).

Hourly study of the spectral relative attenuation of UV highlighted the fact that in the afternoon, location C (southern point of the skylight) presented higher values in winter (50%) at 300 nm.

Relative attenuation was higher in the UV range in the morning in winter at locations within the ruins, with the lowest values at noon. Within the UV range, from 320 to 370 nm, the relative attenuation was very low at all times and locations. In the UVB range, the highest mean UVB and UVA band relative attenuation (20%) was within the ruins (locations D and E) in the morning in both seasons. The relative attenuation of the UVA band was quite constant throughout the museum in both seasons but lower than that of the UVB band, since it is in the range of 0–3%. The highest mean relative attenuation of the VIS and NIR bands also occurred within the ruins (locations C and D) and decreased from morning to afternoon, but with lower relative attenuation values than VIS in the NIR range (morning 35% and afternoon 15%) (morning 50–65% and afternoon 30%).

The comparison of UV spectral relative attenuation for the skylight with and without the water sheet showed that at the 9:00 a.m. solar hour and solar noon, the relative attenuation was generally higher through the empty skylight (day 25-7), except for location E at noon and in the afternoon for locations D and E, since relative attenuation was higher from 380 nm when the skylight was covered by the water sheet.

Measurement of relative attenuation has been found to be a useful tool in the preventive conservation of cultural heritage since this measure is affected by the entire specific architectural and decorative structure of the museum. Architecturally, the water layer that covers the skylight increases the relative attenuation of the solar rays that affect the skylight. Work in progress is currently applying statistical comparison methods to reinforce the results.

## Figures and Tables

**Table 1:** Comparison of specifications of spectrometers used [35].

	HR4000CG-UV-NIR (Ocean Insight)	FLAME-S-UV-VIS (Ocean Insight)
Detector	Linear silicon CCD array	Linear silicon CCD array
Entrance slit	5 µm	25 µm
Grating	#HC1	#1
Pixels	3648	2048
Integration time	4 ms–20 s	1 ms–65 s
Optical resolution	0.47 nm FWHM (typical)	1.33 nm FWHM (typical)
Wavelength range	200–1100 nm	200–850 nm
Input fiber connector	SMA 905	SMA 905
Signal-to-noise ratio	300:1 (full signal)	250:1 (full signal)
Stray light	<0.05% at 600 nm; <0.10% at 435 nm	
Calibration uncertainty	10%	10%
Wavelength step	0.10 nm	0.10 nm

**Table 2:** Solar zenith angle (SZA), in degrees, for the studied periods.

	9 h	12 h	15 h
	(Morning)	(Noon)	(Afternoon)
25/07/2019	44.70	19.72	41.68
30/07/2019	44.84	19.94	41.80
14/01/2020	75.32	60.97	72.74

**Table 3:** Values of mean relative attenuations for the UVB and UVA bands in summer at three times of day.

	Relative Attenuations				
	A	B	C	D	E
9 solar h UVB	0.138	0.130	0.148	0.195	0.151
9 solar h UVA	0.018	0.016	0.026	0.029	0.024
Solar noon UVB	0.055	0.055	0.079	0.081	0.058
Solar noon UVA	0.010	0.010	0.041	0.039	0.012
15 solar h UVB	0.073	0.071	0.075	0.075	0.075
15 solar h UVA	0.013	0.012	0.016	0.016	0.014

**Table 4:** Values of the mean relative attenuations for the UVB and UVA bands in winter at three times of day.

	Relative Attenuations				
	A	B	C	D	E
9 solar h UVB	0.143	0.104	0.132	0.188	0.163
9 solar h UVA	0.022	0.018	0.032	0.022	0.020
Solar noon UVB	0.061	0.057	0.072	0.083	0.062
Solar noon UVA	0.011	0.010	0.035	0.039	0.012
15 solar h UVB	0.076	0.067	0.075	0.082	0.081
15 solar h UVA	0.010	0.011	0.013	0.016	0.011

**Table 5:** Mean relative attenuation values for the VIS and NIR bands in summer and at 3 times of day.

	Relative Attenuations				
	A	B	C	D	E
9 solar h VIS	0.004	0.004	0.672	0.570	0.102
9 solar h NIR	0.009	0.011	0.417	0.300	0.040
Solar noon VIS	0.006	0.003	0.448	0.456	0.023
Solar noon NIR	0.012	0.020	0.226	0.206	0.018
15 solar h VIS	0.219	0.010	0.034	0.357	0.018
15 solar h NIR	0.076	0.006	0.025	0.144	0.016

**Table 6:** Values of the average relative attenuations for the VIS and NIR bands in winter at 3 times of day.

	Relative Attenuations				
	A	B	C	D	E
9 solar h VIS	0.062	0.052	0.143	0.157	0.116
9 solar h NIR	0.307	0.323	0.274	0.237	0.215
Solar noon VIS	0.053	0.033	0.840	0.251	0.302
Solar noon NIR	0.256	0.232	0.770	0.234	0.319
15 solar h VIS	0.006	0.002	0.004	0.006	0.007
15 solar h NIR	0.007	0.006	0.006	0.006	0.008

**Table 7:** Values of the spectral relative attenuations for the UV band for the three interior locations for the two skylights without (25-7) and with water (30-7) at 9 a.m. solar time.

	Relative Attenuations					
nm	C 25-7	C 30-7	D 25-7	D 30-7	E 25-7	E 30-7
300	0.439	0.405	0.648	0.502	0.403	0.392
310	0.124	0.129	0.194	0.149	0.132	0.156
320	0.037	0.041	0.050	0.041	0.041	0.033
330	0.016	0.016	0.022	0.020	0.016	0.015
340	0.016	0.014	0.021	0.019	0.016	0.016
350	0.015	0.014	0.020	0.020	0.016	0.014
360	0.015	0.014	0.021	0.014	0.014	0.013
370	0.011	0.014	0.015	0.014	0.014	0.009
380	0.024	0.019	0.019	0.020	0.020	0.018
390	0.082	0.050	0.058	0.053	0.066	0.050
400	0.159	0.109	0.225	0.163	0.139	0.118

**Table 8:** Values of the spectral relative attenuations for the UV band for the three interior locations for the two skylight states (25-7 without water and 30-7 with water) at solar noon.

	Relative Attenuations					
nm	C 25-7	C 30-7	D 25-7	D 30-7	E 25-7	E 30-7
300	0.368	0.318	0.377	0.321	0.275	0.305
310	0.070	0.061	0.074	0.063	0.050	0.059
320	0.023	0.017	0.024	0.020	0.017	0.016
330	0.011	0.009	0.012	0.010	0.008	0.010
340	0.012	0.012	0.013	0.011	0.008	0.011
350	0.011	0.012	0.014	0.010	0.008	0.010
360	0.011	0.011	0.012	0.009	0.008	0.010
370	0.009	0.008	0.010	0.008	0.007	0.009
380	0.053	0.046	0.050	0.034	0.013	0.039
390	0.273	0.245	0.257	0.168	0.030	0.202
400	0.432	0.399	0.404	0.294	0.069	0.328

**Table 9:** Values of the spectral relative attenuations for the UV band for the three interior locations for the two skylight states (25-7 without water and 30-7 with water) at 15 solar hours.

	Relative Attenuations					
nm	C 25-7	C 30-7	D 25-7	D 30-7	E 25-7	E 30-7
300	0.350	0.316	0.340	0.316	0.304	0.369
310	0.057	0.072	0.065	0.071	0.061	0.059
320	0.019	0.019	0.020	0.024	0.019	0.025
330	0.009	0.009	0.008	0.012	0.009	0.012
340	0.010	0.007	0.010	0.012	0.009	0.010
350	0.009	0.010	0.009	0.010	0.008	0.012
360	0.007	0.007	0.008	0.011	0.010	0.008
370	0.008	0.009	0.008	0.008	0.009	0.008
380	0.016	0.016	0.015	0.014	0.013	0.016
390	0.050	0.046	0.050	0.070	0.032	0.072
400	0.093	0.077	0.106	0.206	0.069	0.151
